# A technique to measure tidal volume during noninvasive respiratory support by continuous-flow helmet CPAP

**DOI:** 10.1007/s10877-023-01034-0

**Published:** 2023-06-17

**Authors:** Andrea Coppadoro, Giacomo Bellani, Giuseppe Foti

**Affiliations:** 1 Department of Anesthesia and Intensive care, San Gerardo Hospital, Fondazione IRCCS San Gerardo dei Tintori, Monza, Italy; 2 Centre for Medical Sciences - CISMed, University of Trento, Trento, Italy; 3 Department of Anesthesia and Intensive Care, Santa Chiara Regional Hospital, APSS Trento, Trento, Italy; 4 Department of Medicine and Surgery, University of Milan-Bicocca, Via Cadore 48, Monza, MB Italy

**Keywords:** Noninvasive respiratory support, Continuous flow Helmet CPAP, Acute hypoxemic respiratory failure, Tidal volume respiratory monitoring, Spontaneous breathing

## Abstract

**Purpose:**

The coronavirus disease 2019 (COVID-19) pandemic has promoted the use of helmet continuous positive airway pressure (CPAP) for noninvasive respiratory support in hypoxic respiratory failure patients, despite the lack of tidal volume monitoring. We evaluated a novel technique designed to measure tidal volume during noninvasive continuous-flow helmet CPAP.

**Methods:**

A bench model of spontaneously breathing patients undergoing helmet CPAP therapy (three positive end-expiratory pressure [PEEP] levels) at different levels of respiratory distress was used to compare measured and reference tidal volumes. Tidal volume measurement by the novel technique was based on helmet outflow-trace analysis. Helmet inflow was increased from 60 to 75 and 90 L/min to match the patient’s peak inspiratory flow; an additional subset of tests was conducted under the condition of purposely insufficient inflow (i.e., high respiratory distress and 60 L/min inflow).

**Results:**

The tidal volumes examined herein ranged from 250 to 910 mL. The Bland‒Altman analysis showed a bias of -3.2 ± 29.3 mL for measured tidal volumes compared to the reference, corresponding to an average relative error of -1 ± 4.4%. Tidal volume underestimation correlated with respiratory rate (rho = .411, p = .004) but not with peak inspiratory flow, distress, or PEEP. When the helmet inflow was maintained purposely low, tidal volume underestimation occurred (bias − 93.3 ± 83.9 mL), corresponding to an error of -14.8 ± 6.3%.

**Conclusion:**

Tidal volume measurement is feasible and accurate during bench continuous-flow helmet CPAP therapy by the analysis of the outflow signal, provided that helmet inflow is adequate to match the patient’s inspiratory efforts. Insufficient inflow resulted in tidal volume underestimation. In vivo data are needed to confirm these findings.

## Introduction

Noninvasive respiratory support is indicated for respiratory failure treatment when patients do not improve by standard oxygen supplementation (i.e., nasal prongs or oxygen mask) but tracheal intubation is not needed or contraindicated. Noninvasive respiratory support can be delivered by several devices, such as large-bore nasal cannulas, a tight face mask, or a helmet; while it is indicated to treat patients affected by cardiogenic pulmonary edema or chronic obstructive pulmonary disease exacerbation, it should be used with caution during acute hypoxemic respiratory failure [1].

Noninvasive respiratory support by head helmet is particularly useful for acute hypoxemic respiratory failure since it is associated with lower mortality and failure rates than high flow oxygen or face masks [2–5]. Helmet, as a face mask, can be used either to provide continuous positive airway pressure (CPAP) if it is connected to a continuous-flow generator or to deliver noninvasive positive pressure ventilation when it is connected to a ventilator [6]. During the recent coronavirus disease 2019 (COVID-19) pandemic, several advantages of continuous-flow helmet CPAP were described: high oxygen delivery (up to 100%), relatively high positive end-expiratory pressure (PEEP) levels (up to 10–15 cmH_2_O), device tolerability for prolonged continuous therapy (several consecutive days), possibility of awake prone position, simple equipment with no need for a ventilator, and use outside the intensive care unit [7–10]. However, helmet CPAP presents some limitations compared to ventilator-driven mask CPAP, such as the impossibility of use in claustrophobic patients, the need for a high amount of gas (i.e., usually 60–90 L/min) to maintain an adequate helmet flow to match the patient’s peak inspiratory flow and the lack of tidal volume monitoring.

Preserving spontaneous breathing by noninvasive support presents several advantages as well as some risks: for example, high tidal volumes are associated with worse outcomes, especially during controlled mechanical ventilation [11, 12]. Excessive lung stretch associated with high tidal volumes and elevated transpulmonary pressures could lead to patient self-inflicted lung injury, further exacerbating lung disease and leading to noninvasive treatment failure [13].

We reasoned that continuous monitoring of tidal volume would be a relevant improvement during helmet CPAP therapy, allowing clinicians to prevent lung injury associated with excessive tidal volumes, such as when respiratory support is provided by a face mask coupled with a ventilator. Ideally, the monitoring device should be embedded in the continuous-flow generator. First, we claim that gas outflow from the helmet is influenced by the patient’s respiratory activity, being decreased during inspiration and increased during expiration, despite a constant flow being blown into the helmet (inflow). Hence, we reasoned that at end-expiration (i.e., no patient’s effort) inflow and outflow are equal; when inspiration begins, a fraction of the inflow is diverted into the airways, resulting in a reduction of the outflow corresponding to the inspiratory tidal volume. Conversely, the outflow increases during expiration as the sum of the patient’s expiratory flow and the constant inflow. We hypothesized that analyses of the inflow and outflow waveforms would allow a reliable measurement of the tidal volume generated by a patient simulator. Second, we reasoned that an inspiratory flow higher than the inflow would temporarily interrupt the outflow due to diversion of the entire inflow into the airways. Thus, we hypothesized that tidal volumes would be underestimated if the patient’s peak inspiratory flow was higher than the helmet inflow.

## Methods

**Fig. 1:**
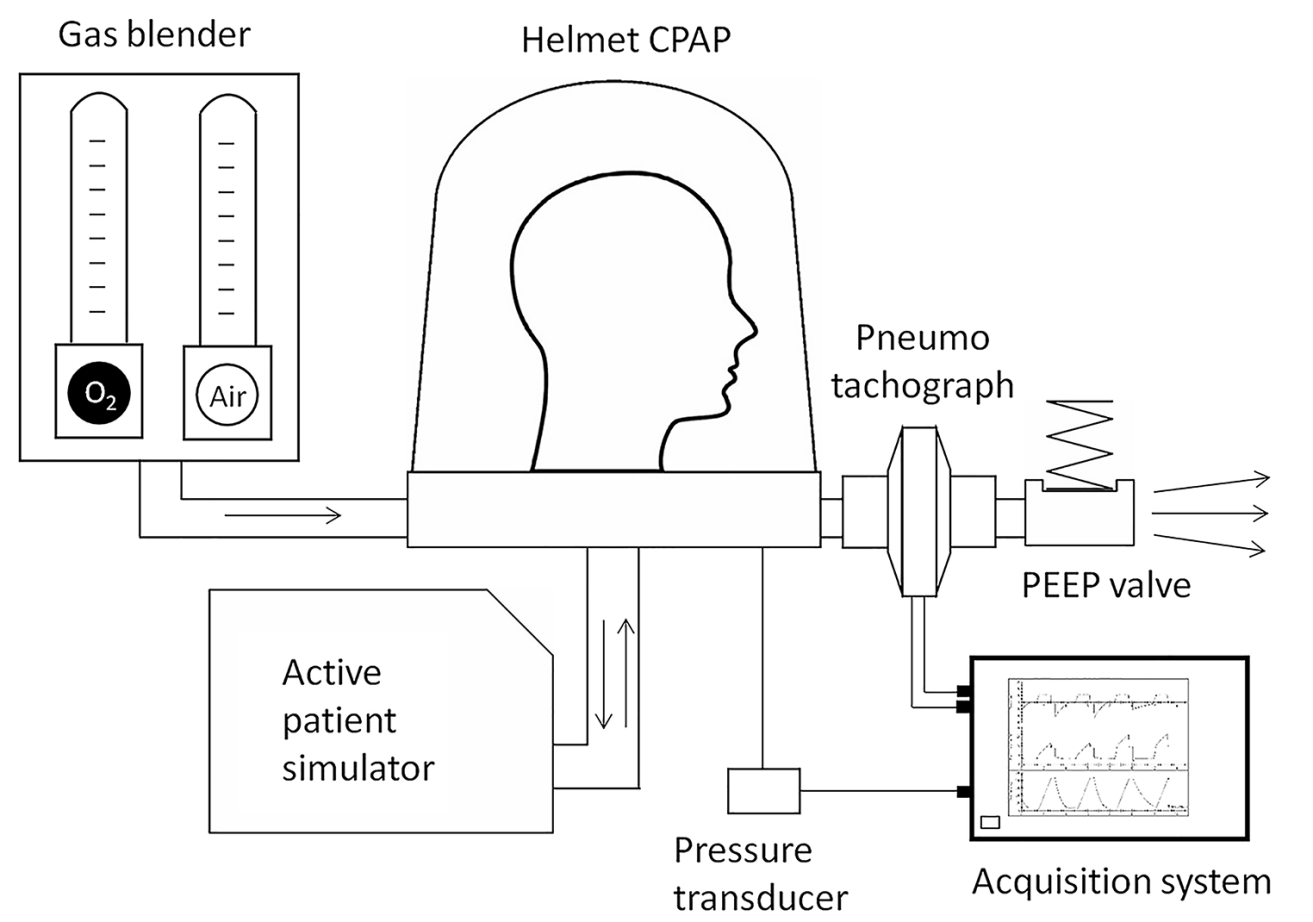
Schematic representation of the noninvasive continuous-flow helmet CPAP respiratory circuit. A constant gas flow (either 60, 75 or 90 L/min) entered the helmet inlet port and escaped through the outlet port while active breathing efforts were simulated, and a mechanical PEEP valve maintained a constant pressure into the helmet (CPAP). A pneumotachograph placed between the helmet and the PEEP valve recorded the outflow

The bench model designed to measure tidal volumes was based on a standard continuous-flow helmet CPAP system: a compressed gas flow regulator (Flowmeter, Levate, Italy) generating the inflow was connected to a helmet (DimAir, Dimar, Mirandola, Italy) equipped with three different mechanical nonadjustable PEEP valves (5, 10, 15 cm H_2_O) from the same manufacturer (Respironics Novametrix, Wallingford, CT, USA). The helmet was worn onto a head model and secured over a board; the head model was connected to a patient simulator (ASL 5000, Ingmar Medical, Pittsburg, PA, USA). A pneumotachograph (A. Fleisch, Switzerland) was positioned at the outlet port before the PEEP valve: the flow waveforms of the gas passing through the PEEP valve were recorded by an analog acquisition system (PowerLab, ADInstruments) and analyzed with dedicated software (Labchart, ADInstruments) to measure tidal volumes (Fig. 1). The possible occurrence of a pneumotachograph drift error was prevented by zero flow calibration every 5 min, if deemed necessary. Zeroing was performed with the appropriate LabChart command after temporary disconnection of the pneumotachograph from the circuit.

Pressure levels within the helmet were also continuously recorded to verify the actual peep levels by a standard invasive pressure transducer (TruWave, Edwards, Germany) connected to the helmet through a plastic tube. The patient simulator was set as follows: 60 ml/cmH_2_O respiratory system compliance; 3 cmH_2_O/L/s resistance; a heat and moisture exchanger filter connected in series to the respiratory circuit to protect the simulator; 10% inspiratory rise time, 5% inspiratory hold time, 10% inspiratory release time; and 10% pause time with passive expiration. The tidal volume generated by the simulator was considered the standard reference and recorded for comparisons; the simulated patient’s peak inspiratory flow was also recorded. Simulated respiratory efforts were managed by variations in inspiratory muscle pressure (pMusc) and respiratory rate (RR), as detailed below.

### Signal processing and tidal volume measurement

**Fig. 2:**
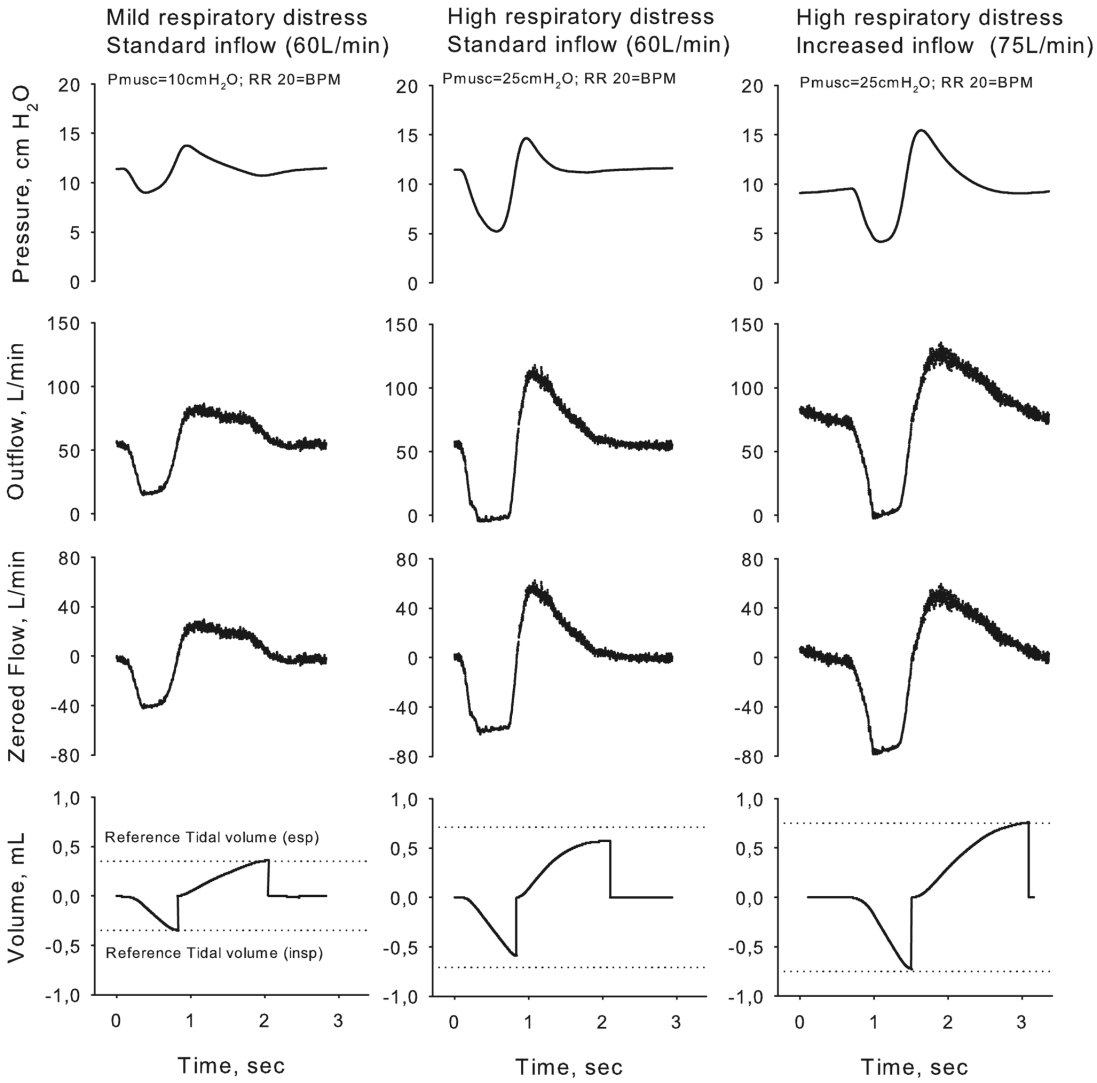
Exemplary plots of pressure within the helmet (first row) and recorded outflow trace (second row) acquired during a simulated respiratory act. Zero offset of the outflow trace was adjusted to obtain net outflow oscillations (third row), resulting in tidal volumes by integral calculation (fourth row). Tidal volume measurements were accurate when inflow was adequate relative to respiratory distress (mild distress and standard flow, left column; high distress and increased distress, right column). Under conditions of high distress and inadequate flow, tidal volumes were underestimated compared to the reference (central column)

Outflow waveforms were acquired at 100 Hz (Fig. 2, second row) and processed to calculate tidal volumes. First, the value of the constant helmet inflow was subtracted from the recorded outflow waveform, resulting in a waveform (named “zeroed outflow”) with a shape identical to the original but with a different off-set to obtain a null value at end expiration (e.g., 60 L/min outflow recorded at the PEEP valve – 60 L/min inflow from the compressed gas regulator; Fig. 2, third row). Second, zeroed outflow waveforms were integrated and reset at each cycle, resulting in an inspiratory and an expiratory tidal volume (Fig. 2, fourth row). Off-set of the zeroed outflow waveform was fine-tuned to equalize inspiratory and expiratory volumes. The average tidal volume obtained during three respiratory cycles was considered for analyses.

### Bench tests

The first set of tests (adequate inflows) was performed by maintaining the inflow at a level higher than the patient’s peak inspiratory flow for any inspiratory effort. Mild-moderate inspiratory efforts (pMusc 10 and 15 cmH_2_O) were studied with 60 L/min helmet inflow, resembling clinical practice. Tidal volumes were measured at three PEEP levels (5, 10, 15 cmH_2_O) under different RR combinations (15, 20, 25 breaths per minutes). Strong inspiratory efforts (pMusc 20 and 25 cmH_2_O) were studied at 75 and 90 L/min inflows to mimic a condition in which inflow is increased to match the patient’s inspiratory effort. The three aforementioned PEEP levels were studied at different RR combinations (15, 20, 25 breaths per minutes for pMusc 20 cmH_2_O; 20, 25 breaths per minutes for pMusc 25 cmH_2_O).

In the second set of tests (insufficient inflows), we studied the patterns of strong inspiratory effort at a standard helmet inflow (60 L/min) to test how the system accuracy would be affected in conditions of insufficient inflow to match the patient’s peak inspiratory flows. In such conditions, during part of the inspiration, the inflow is completely diverted into the lung simulator due to the strong inspiratory effort, and the outflow drops to zero; lower accuracy and underestimation of tidal volume were therefore expected during this set of tests.

### Statistical analysis

Data are presented as the mean ± standard deviation, as not otherwise specified. The normality of the collected variables was assessed using the Kolmogorov‒Smirnov test. Correlations between normal variables were evaluated by Pearson’s correlation coefficient; otherwise, Spearman’s rho was used. Agreement between the reference tidal volumes and the volumes measured with the novel technique was evaluated by the Bland‒Altman plot.[14] Variables collected in the same conditions of respiratory effort but with different helmet inflows were compared by the Mann‒Whitney test. Artworks were created using SigmaPlot 11.0 (Systat software, Palo Alto CA).

## Results

A total of 63 combinations of PEEP, pMusc, RR, and helmet inflow were included in the analysis. Peak inspiratory flows ranged from 39 to 112 L/min; tidal volumes ranged from 250 to 910 mL.

### Adequate inflow tests

A total of 18 combinations were studied in the mild-moderate inspiratory effort tests at standard (60 L/min) inflows; peak inspiratory flow ranged from 39 to 73 L/min, and reference tidal volumes ranged from 250 to 610 mL. Reference tidal volumes and measured volumes were tightly correlated (r = .990 p < .001); the Bland‒Altman analysis showed a bias of -9.2 ± 27.4 mL, corresponding to an average percent error of -2.3 ± 3.4% (range − 9.3 to 2.3%).

A total of 30 combinations were studied in the strong inspiratory effort tests at increased (75 and 90 L/min) inflows; peak inspiratory flow ranged from 74 to 112 L/min, and reference tidal volumes ranged from 475 to 910 mL. Reference tidal volumes and measured volumes were tightly correlated (r = .969 p < .001). The Bland‒Altman analysis showed a bias of 0.3 ± 69.2 mL, which was not different from the mild-moderate condition (p = .189 by Mann‒Whitney Test), corresponding to an average percent error of 0.2 ± 4.8% (range − 9.1 to 8.9%).

**Fig. 3:**
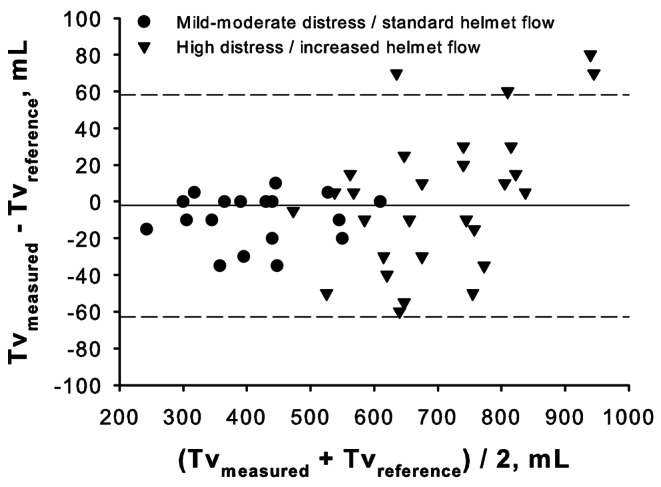
Bland‒Altman plot for tidal volume values obtained by the novel measurement method in comparison with reference values during simulated continuous-flow helmet CPAP therapy under different combinations of PEEP, respiratory rate and inspiratory effort. Helmet inflow was maintained at a sufficient level with respect to simulated respiratory distress: a standard level of 60 L/min was set during mild-moderate respiratory distress simulations (pMusc 10 and 15 cmH_2_O, circles), while increased flows (75 and 90 L/min) were set in cases of high respiratory distress (pMusc 20 and 25 cmH_2_O, triangles)

Pooling together data obtained from the adequate inflow tests, the Bland‒Altman analysis showed a bias of -3.2 ± 29.3 mL, corresponding to an average percent error of -1 ± 4.4% (Fig. 3). In univariate analyses, tidal volume underestimation correlated with RR (rho = .411, p = .004) but not with peak inspiratory flow (p = .537), pMusc (p = .989), or PEEP (p = .382).

### Insufficient inflow

**Fig. 4:**
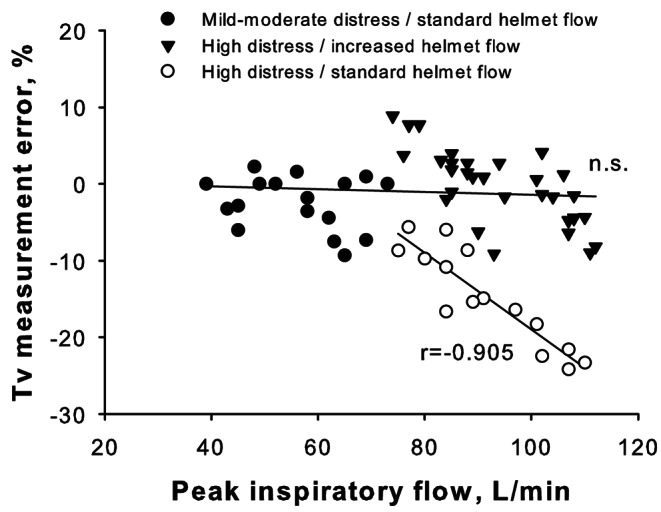
Tidal volume measurement error was lower than ± 10% during continuous-flow helmet CPAP therapy, provided that the inflow was maintained at a sufficient level with respect to the patient’s respiratory distress (filled symbols). A standard helmet inflow (60 L/min), resulting lower than peak inspiratory flow in the presence of high respiratory distress (pMusc 20 and 25 cmH_2_O, empty circles), led to underestimation of tidal volumes; the higher the peak inspiratory flow, the higher the underestimation error (r=-.905 p < .001)

A total of 15 combinations were studied in the strong inspiratory effort tests at standard (60 L/min) inflow, a value purposely lower than inspiratory flow, to verify the hypothesis that tidal volume is underestimated when inflow does match the patient’s peak inspiratory effort. Reference tidal volumes ranged from 480 to 765 mL, while peak inspiratory flows exceeded the inflow ranging from 75 to 110 L/min. Reference tidal volumes were significantly correlated with measured volumes (r = .882 p < .001). However, the Bland‒Altman analysis showed a bias of -93.3 ± 83.9 mL, which was higher than the mild-moderate condition with 60 L/min inflow (p < .001 by Mann‒Whitney Test). The corresponding average relative error showed a consistent tidal volume underestimation (-14.8 ± 6.3% compared to reference, range − 24.2 to 5.6%, open circles in Fig. 4), closely related to high peak inspiratory flows (r=-.905 p < .001), pMusc (rho − 0.819 p < .001), RR (rho − 0.730 p = .002), but not PEEP (p = .841).

## Discussion

In the present bench study, we showed that it is possible to measure tidal volumes during Helmet CPAP by simply monitoring the outlet port of the helmet. Several combinations of PEEP, respiratory rate and inspiratory effort were investigated, mimicking a wide range of clinical possibilities. Tidal volume measurements were quite accurate (error below ± 10%) along a broad spectrum of simulations, provided that the helmet inflow was adjusted to the level of the patient’s inspiratory distress. Higher patient peak inspiratory flows coupled with insufficient inflows were associated with tidal volume underestimation.

The study was designed as a proof-of-concept to show the feasibility and effectiveness of a simple technique to measure patients’ tidal volumes during helmet CPAP noninvasive respiratory support. The principle at the basis of the measurement is quite simple: inspiratory tidal volume is measured as the inlet volume not flowing out of the helmet, and expiratory volume is measured as the outlet volume in excess on top of the expected continuous outflow. The prerequisite to obtain a reliable measurement is to adjust the inflow to the patient’s inspiratory needs, as clinically indicated when strong inspiratory efforts cause relevant helmet pressure oscillations, avoiding (or minimizing) phases of zero outflow during inspiration. When outflow is reduced to zero, the amount of patient’s inspiratory flow exceeding the inflow is not detectable at the outlet port, leading to an underestimation of the measured tidal volume. It should be noted that in the case of inspiratory flow exceeding the inflow, the patient can still receive flow from the helmet compression volume, which cannot be computed by a simple outflow trace measurement. Such a limitation is intrinsic to the technique but can be easily overcome: when inspiratory effort is such that inflow becomes insufficient (as detected by tidal interruption of outflow), the clinician should increase the inflow to reduce helmet pressure swings; additionally, correct tidal volume measurements will be restored.

Tidal volume monitoring is a relevant piece of information during noninvasive respiratory support to prevent the risk of excessively large tidal volumes associated with noninvasive treatment failure and further lung damage. In common clinical practice, tidal volume is not measured during helmet CPAP therapy and is possible during mask CPAP therapy only when using a ventilator. Therefore, several tidal volume measurement techniques have been studied in the past, and others are now emerging as novel noninvasive devices. Bedside noninvasive measurement of tidal volumes by inductive plethysmography has been known for many years, but clinical application is limited to pediatric settings [15, 16]. Improved plethysmography techniques, such as optoelectronic or fiber optic, were studied but remained at a research level; thoracic impedance or exhaled gas temperature were investigated in recent years with promising results and began to be used in the clinical scenario [17–21]. Recently, a modified ventilator connected to a helmet equipped with an intentional leak port was used to measure tidal volumes during CPAP therapy with good results [22].

The main advantage of the described technique is its simplicity: it is applicable to a standard continuous-flow helmet CPAP system requiring only a pneumotachograph and a signal processing unit, which could be easily integrated into a continuous-flow generator machine. The trend toward an underestimation error will result in a high positive predictive value for the detection of excessive tidal volume during noninvasive respiratory support: if the tidal volume measured by the system is elevated, the actual tidal volume would be similar or higher, revealing a condition of potential harm for the patient due to self-inflicted lung injury. The tested helmet inflow levels (60, 75 and 90 L/min) resemble levels typically used for patient care: a standard inflow level of 60 L/min is commonly used for the care of mild-distress patients, while distressed patients may require inflow levels up to 90 L/min. Patient comfort is generally not affected by higher inflows because the gas freely escapes through the PEEP valve; however, increased noise may be noted [23]. The three tested PEEP levels (5, 10 and 15 cm H_2_O) covered the most common helmet use; the effectiveness of the proposed tidal volume monitoring system at the highest PEEP level suggests that PEEP is not a limiting factor.

However, the presented data also show limitations, mainly due to the bench nature of the study: in vivo tests are lacking, especially the presence of variable leaks, which could be a limitation; signal processing needs to be automated; and confounding factors frequently present in the clinical scenario, such as the presence of leaks or the use of different PEEP valves, need to be evaluated.

## Conclusions

In the present proof-of-concept bench study, we showed that tidal volume measurement based on the analysis of the outflow signal is feasible and accurate during continuous-flow helmet CPAP therapy, provided that helmet inflow is maintained at a sufficient level to match the patient’s peak inspiratory flow. Insufficient inflow resulted in tidal volume underestimation. In vivo data are needed to confirm such findings.
